# Einfluss des Rauchens auf den Gastrointestinaltrakt: Spektrum der Befunde

**DOI:** 10.1007/s00117-022-01017-3

**Published:** 2022-06-23

**Authors:** Sabine Popp, Thomas Mang, Martina Scharitzer

**Affiliations:** Universitätsklinik für Radiologie und Nuklearmedizin, Medizinische Universität Wien, Währinger Gürtel 18–20, 1090 Wien, Österreich

**Keywords:** Tabak, Nikotin, Kanzerogene, Karzinom, Inflammation, Tobacco, Nicotine, Carcinogens, Neoplasms, Inflammation

## Abstract

**Hintergrund:**

Nikotin ist ein hochwirksames Suchtgift, das bei regelmäßiger Einnahme chronische oder unheilbare Erkrankungen und somit eine eingeschränkte Lebensqualität zur Folge haben kann.

**Fragestellung:**

Das Ziel dieser Übersichtsarbeit besteht darin, mögliche gesundheitliche Folgen des Rauchens auf den Gastrointestinaltrakt aufzuzeigen und einen Überblick über raucherassoziierte neoplastische und nichtneoplastische gastrointestinale Erkrankungen zu geben.

**Material und Methode:**

Anhand einer ausführlichen Literaturrecherche wird der aktuelle Wissensstand zu raucherassoziierten Folgen auf den Gastrointestinaltrakt dargestellt.

**Ergebnisse:**

Rauchen ist ein wesentlicher Risikofaktor für die Entstehung neoplastischer und nichtneoplastischer Erkrankungen des gesamten Gastrointestinaltrakts. Diese weisen in der radiologischen Bildgebung allerdings keine spezifischen, raucherassoziierten Merkmale auf.

**Schlussfolgerung:**

Die Kenntnis einer Raucheranamnese sowie möglicher Auswirkungen von Nikotin auf den Gastrointestinaltrakt können in der radiologischen Bildinterpretation hilfreich sein sowie die diagnostische Entscheidungsfähigkeit und Genauigkeit verbessern.

## Abstract (en)

**Background:**

Nicotine is a highly addictive drug that, if consumed regularly, can result in chronic or incurable diseases and a reduced quality of life.

**Objectives:**

The aim of this review is to describe the possible health consequences of smoking on the gastrointestinal tract and to provide an overview of related neoplastic and nonneoplastic gastrointestinal diseases.

**Materials and methods:**

Based on an extensive literature review, the current knowledge about smoker-associated diseases of the gastrointestinal tract is summarized.

**Results:**

Smoking is a significant risk factor for the development of neoplastic and nonneoplastic diseases within the entire gastrointestinal tract. However, they are not associated with specific, smoking-associated radiologic imaging features.

**Conclusion:**

Knowledge of a smoker’s history and possible consequences of nicotine on the gastrointestinal tract can help in the interpretation of radiological images and improve diagnostic decision-making skills and accuracy.

Tabakrauch hat durch toxische und kanzerogene Inhaltsstoffe neben Lungenerkrankungen auch eine Fülle von unterschiedlichen Effekten auf den Gastrointestinaltrakt. Er induziert neoplastische und entzündliche Veränderungen, beeinflusst das Mikrobiom und die Entstehung von Autoimmunerkrankungen (Tab. 1). Im Folgenden werden diese Effekte anhand der jeweiligen Krankheitsbilder in den einzelnen Abschnitten des Gastrointestinaltrakts besprochen und die charakteristischen bildgebenden Merkmale der einzelnen Erkrankungen anhand von Bildbeispielen erläutert. Hierbei ist allerdings zu beachten, dass es in der radiologischen Bildgebung keine für Raucher spezifischen morphologischen Merkmale dieser Krankheitsbilder im Vergleich zu denen für Nichtraucher gibt.

**Table 1:** 

Organ	Erkrankung	Folgen
*Negative Auswirkungen*		
Oropharynx	Parodontitis	Höhere Inzidenz, aggressivere Verläufe
	Karzinom	Höhere Inzidenz
Ösophagus	GERD	Geringer Tonus UES, verlängerte Säure-Clearance-Zeit
	Karzinom	Höhere Inzidenz
Magen	Peptisches Ulkus	Höhere Inzidenz, häufigere *H.-pylori*-Infektion, langsamere Heilungsrate, höheres Rezidivrisiko
	Karzinom	Höhere Inzidenz
Darm	M. Crohn	Schwererer Krankheitsverlauf, höhere Rezidivrate
	Neoplasien	Höhere Inzidenz von Karzinomen, Adenomen und serratierten Polypen
Leber	Fibrose	Erhöhte Insulinresistenz?
	HCC	Höhere Inzidenz
Pankreas	Pankreatitis	Höhere Inzidenz der akuten und chronischen Pankreatitis, schwererer Verlauf bei alkoholisch chronischer Pankreatitis
	Karzinom	Höhere Inzidenz
*Positive Auswirkungen*		
Darm	Colitis ulcerosa	Geringere Inzidenz bei neu diagnostizierter CU, kein Unterschied im Krankheitsverlauf
	Zöliakie	Geringere Inzidenz
	Pouchitis	Unterschiedliche Studiendaten
Leber	PSC	Geringere Inzidenz

*CU* Colitis ulcerosa, *GERD* gastroösophageale Refluxkrankheit, *HCC* hepatozelluläres Karzinom, *PSC* primär sklerosierende Cholangitis, *UES* Unterer Ösophagussphinkter

Der Tabakrauch stellt ein komplexes chemisches Gemisch aus u. a. Nikotin, Aldehyden, polyzyklischen aromatischen Kohlenwasserstoffen, Nitrosaminen und Schwermetallen dar. Im Tabak und Tabakrauch finden sich über 70 Karzinogene, inklusive tabakspezifischer Nitrosamine. Von diesen wurden NNK (Nikotin-Nitrosamin-Keton) und NNN (N-Nitroso-Nornikotin) von der IARC (International Agency for Research on Cancer) als Gruppe-1-Karzinogene eingestuft, wobei NNK als wesentlicher Risikofaktor für Lungenkarzinome und NNN für die Entstehung z. B. von Mundhöhlen- und Ösophaguskarzinomen zugeordnet wird [1]. Tabakrauch enthält neben dem für die Tabakabhängigkeit verantwortlichen Nikotin zahlreiche weitere Zusatzstoffe, die über die Bildung von freien Radikalen und über die Induktion von chronischen Entzündungen in weiterer Folge zur Entstehung von Karzinomen führen können [2]. Die nachteiligen Auswirkungen des Rauchens auf die Lunge und das Herz-Kreislauf-System sind vielfach beschrieben. Hingegen sind die Folgen auf den Gastrointestinaltrakt noch nicht ganz geklärt. Beim Rauchen kommt es nicht nur zu einer Inhalation, sondern auch zum Verschlucken von Zigarettenrauch mit möglichen direkten Folgen auf den gesamten Gastrointestinaltrakt.

## Effekt von Rauchen auf das intestinale Mikrobiom

In letzter Zeit ist das Mikrobiom des Darms zunehmend in den Fokus gerückt. Unter dem intestinalen Mikrobiom versteht man die Gesamtheit aller im Darm vorhandenen Mikroorganismen. Dazu zählen mehr als 1 × 10^14 Mikroben, die wesentliche physiologische Funktionen wie die Barrierefunktion der Darmwand, den Metabolismus, den Immunstatus sowie das inflammatorische Geschehen beeinflussen [3]. Zahlreiche Studien unterstützen die Hypothese, dass neben der genetischen Prädisposition das intestinale Mikrobiom eine wesentliche Rolle bei der Entstehung von Erkrankungen und Syndromen spielt, die sowohl den Darm als auch andere Organsysteme betreffen. Dazu zählen z. B. entzündliche Darmerkrankungen, kardiovaskuläre Ereignisse, Kollagenosen sowie Veränderungen des Zentralnervensystems (ZNS) und auch die Entstehung von Karzinomen. Das Mikrobiom ist u. a. von genetischen Faktoren und dem Patientenalter abhängig, reagiert zudem sensibel auf orale medikamentöse Therapien wie z. B. Antibiotika sowie auf Ernährungsgewohnheiten und andere Lebensstilfaktoren, besonders auf das Rauchen [3].

Durch verschiedene Mechanismen kann der verschluckte Tabakrauch im Gastrointestinaltrakt zu einer Dysbiose des Mikrobioms führen: Nikotin, die primäre aktive Substanz des Zigarettenrauchs, vermindert durch eine Erhöhung des intestinalen pH-Werts einerseits die Anzahl der *Bacteroides* und erhöht andererseits die Anzahl an *Firmicutes* und Proteobakterien. Zudem ist Zigarettenrauch die wesentliche Expositionsquelle für Aldehyde, wobei Acetaldehyd durch intestinale Bakterien in Ethanol umgewandelt wird, was vorteilhaft für *Bacillus* und *Acinetobacter* ist. Auch die im Tabakrauch enthaltenen polyzyklischen aromatischen Kohlenwasserstoffe, Schwermetalle und toxischen Gase wie z. B. Kohlenmonoxid zeigen einen Einfluss auf das Mikrobiom [3]. Neben der Änderung der mikrobiellen Zusammensetzung und Vielfalt schädigt der Zigarettenrauch zusätzlich die Durchlässigkeit der Darmbarriere im Ileum durch eine Atrophie der Darmzotten sowie eine Störung der „Tight-junction“-Proteine, die als Verbindung zwischen den Epithelzellen für die Dichtheit verantwortlich sind [4].

## Darm-Lungen-Achse

Verglichen mit dem intestinalen Mikrobiom zeigt die Lunge eine deutlich weniger dichte mikrobielle Besiedelung. Die Zusammensetzung des Mikrobioms der Lunge ist u. a. durch Mikroinhalation von Speichel wesentlich von der mikrobiellen Besiedelung des Oropharynx sowie des oberen Respirationstrakts abhängig. Die überwiegenden Bakterienstämme sind dieselben wie im Intestinum, nämlich *Firmicutes* und *Bacteroides*, gefolgt von Proteobakterien und Aktinobakterien [5].

Der Begriff „Darm-Lungen-Achse“ umfasst die Wechselwirkung zwischen der Darmflora und dem Mikrobiom der Atemwege. Beispielsweise können Veränderungen des intestinalen Mikrobioms den Verlauf von Lungenerkrankungen (wie z. B. Asthma) wesentlich beeinflussen. Eine Schädigung der Atemwegsorgane hingegen verursacht gleichermaßen auch eine Dysbalance der Darmflora, die ihrerseits durch eine Aktivierung der Leukozyten zu einer Beeinträchtigung des Lungenparenchyms führen kann. Durch Zufuhr physiologisch günstiger Darmbakterien kann man das Gleichgewicht des Mikrobioms wiederherstellen und so die Leukozytenaktivität sowie die Entzündungsfaktoren reduzieren [6].

## Auswirkungen des Rauchens auf den Gastrointestinaltrakt

### Kopf-Hals-Bereich

#### Kopf-Hals Tumoren

Bösartige Tumoren des Kopf-Hals-Bereichs waren 2018 weltweit die siebthäufigsten Karzinome und machten 3 % aller Karzinome sowie 1,5 % aller mit Krebs assoziierten Todesfälle aus [7].

**Figure 1:**
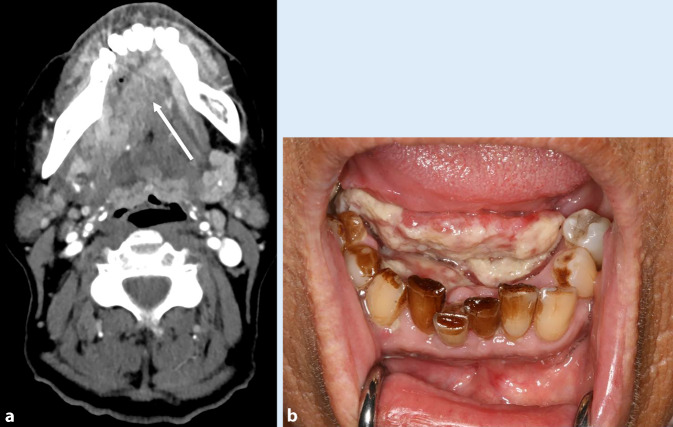

**Figure 2:**
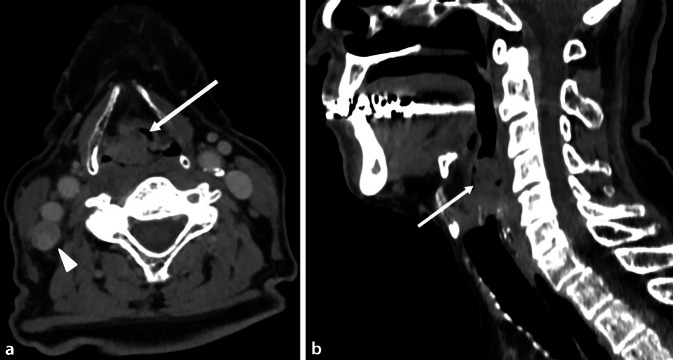

Histologisch sind diese Malignome zum überwiegenden Anteil Plattenepithelkarzinome, wobei Tabak, Alkohol und Infektionen mit dem humanen Papillomvirus (HPV) die wesentlichen Risikofaktoren für deren Entstehung darstellen [8]. Im aktuellen Tabakatlas wurde für Deutschland eine Steigerung des Risikos für die Entstehung von Karzinomen der Mundhöhle und des Rachens in allen Altersgruppen bei Männern um das 10,9Fache und bei Frauen um das 5,1Fache beschrieben (Abb. 1; [9]). Von allen Kopf-Hals-Tumoren ist Rauchen beim Larynxkarzinom für die höchste Risikosteigerung verantwortlich. Je nach Dauer und Intensität des Rauchens steigt das Risiko für Pharynxkarzinome auf das bis zu 10Fache und für Larynxkarzinome auf das bis zu 40Fache im Vergleich zu Personen, die niemals geraucht haben (Abb. 2). Ebenso steigern andere Tabakprodukte, wie z. B. Kautabak, das Risiko eines Plattenepithelkarzinoms, v. a. in der Mundhöhle als auch im Oropharynx um das 2‑ bis 4Fache [10]. Die Beendigung des Rauchens hat einen wesentlichen Einfluss auf das Karzinomrisiko. Bei Personen, die vor mehr als 10 Jahren zu rauchen aufgehört hatten, sank das Risiko für orale und pharyngeale Karzinome um die Hälfte und für Larynxkarzinome um ein Drittel [11].

#### Parodontitis

Parodontitis ist eine multifaktorielle, chronisch-entzündliche Erkrankung, die mit einer Destruktion des Zahnhalteapparats einhergeht. Bekannte Risikofaktoren der Parodontitis sind genetische Faktoren, systemische Erkrankungen, Zahnstein, Zahnfehlstellungen, die zu einer Malokklusion führen sowie eine unzureichende Sanierung kariöser Defekte. Rauchen ist ein wesentlicher Lebensstilfaktor für die Entstehung von Parodontitis. Im Vergleich zu Nichtrauchern weisen Raucher ein 90 % höheres Risiko auf. Des Weiteren treten aggressive Verläufe der Parodontitis bei Rauchern häufiger auf als bei Nichtrauchern [12].

### Ösophagus

#### Gastroösophageale Refluxkrankheit

Ein gastroösophagealer Reflux kann durch langanhaltenden pathologischen Rückfluss von Magensäure in den Ösophagus zu einer gastroösophagealen Refluxerkrankung (GERD) mit klinischen Symptomen und entsprechenden Komplikationen wie Ösophagitis, Strikturen, intestinalen Metaplasien (Barrett-Ösophagus) sowie von Adenokarzinomen führen. GERD hat weltweit eine hohe Prävalenz, die in Europa, den USA und Australien zwischen 9 und 28 % liegt [13].

Rauchen kann einen gastroösophagealen Reflux induzieren, indem es den Ruhetonus des unteren Ösophagussphinkters reduziert. In mehreren Studien wurde ein Absinken des Tonus des unteren Ösophagussphinkters um bis zu 41 % nachgewiesen [14]. Ursächlich für die Reduktion des Tonus dürfte die Blockade von cholinergen Rezeptoren durch Nikotin sein, die zu einer konsekutiven Entspannung der zirkulären Muskelfasern des unteren Ösophagussphinkters führt [13]. Des Weiteren wurde eine verlängerte Säure-Clearance-Zeit beobachtet, offenbar als Folge einer durch das Rauchen verringerten Speichelsekretionsrate und Bikarbonatkonzentration [13].

Nach Beendigung des Rauchens kam es bei normalgewichtigen Personen zu einer Symptomreduktion des gastrointestinalen Refluxes. Bei übergewichtigen Personen zeigte sich jedoch keine Besserung, was durch die ausgeprägte Assoziation zwischen Übergewicht und GERD zu erklären ist [14].

#### Ösophaguskarzinom

**Figure 3:**
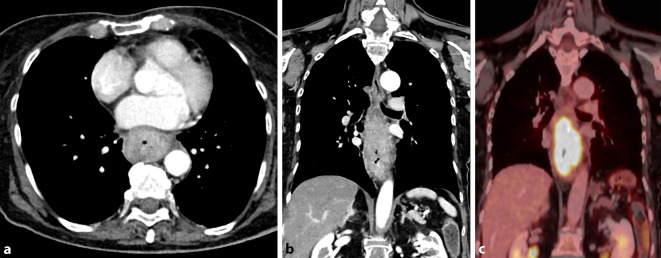

Rauchen führt bei Frauen zu einem 7,8fach und bei Männern zu einem 6,8fach erhöhten Risiko für die Entstehung eines Ösophaguskarzinoms (Abb. 3).

Beim Plattenepithelkarzinom (PEC) besteht eine starke dosisabhängige Beziehung zwischen den Pack Years und dem Krebsrisiko. Dabei sind weniger Zigaretten über einen längeren Zeitraum schädlicher als mehr Zigaretten in einem kürzeren Zeitraum [15]. Darüber hinaus führt auch Passivrauchen zu einem erhöhten PEC-Risiko. Bei ehemaligen Rauchern kommt es zu einem linearen Abfall des Risikos in Abhängigkeit von der Dauer der Tabakabstinenz. Ein positiver Effekt wird bereits nach 5 Jahren erreicht, das Risiko normalisiert sich ab einer Raucherabstinenz von mehr als 20 Jahren [16].

Bei Adenokarzinomen zeigte sich ebenfalls ein dosisabhängiges, moderat erhöhtes Risiko, wobei es zu einem linearen Anstieg des Krebsrisikos abhängig von den Pack Years kommt. In einer umfangreichen Analyse wurde gezeigt, dass das Risiko, ein Adenokarzinom zu entwickeln, doppelt so hoch bei Rauchern und Ex-Rauchern ist wie bei Personen, die nie geraucht haben. Das Risiko für die Entwicklung eines Adenokarzinoms aus einem bestehenden Barrett-Ösophagus wird durch Rauchen erhöht, nicht jedoch das für die Entstehung des Barrett-Ösophagus. Im Gegensatz zum Plattenepithelkarzinom unterscheidet sich bei Adenokarzinomen das Risiko zwischen ehemaligen und aktiven Rauchern kaum. Des Weiteren zeigt sich der positive Effekt der Tabakabstinenz erst deutlich später: Erst ab einem rauchfreien Intervall von mehr als 20 Jahren sinkt das Risiko um etwa 30 % [17].

Die Kombination mit Alkohol potenziert das Risiko für die Entstehung von Plattenepithelkarzinomen bei Rauchern auf das mehr als 20Fache [17]. Bei Adenokarzinomen ist hingegen lediglich das Rauchen mit einem erhöhten Risiko vergesellschaftet, nicht jedoch der Alkoholkonsum. Die sinkende Inzidenz der PEC in westlichen Ländern dürfte auf den Rückgang von Tabak- und Alkoholkonsum zurückzuführen sein [9, 17].

### Magen

#### Peptische Ulzera

Die Hauptrisiken für die Entstehung peptischer Ulzera sind Infektionen mit *Helicobacter pylori* und der Langzeitgebrauch von nichtsteroidalen Antirheumatika (NSAR). Rauchen erhöht das Risiko einer Infektion mit *H. pylori*, verlangsamt die Heilung peptischer Ulzera und steigert das Rezidivrisiko. Ursächlich dafür sind die durch das Rauchen erhöhte Pepsinproduktion und der verminderte Schutz der Magenschleimhaut aufgrund geringerer Durchblutung sowie eine geringere Schleim- und Natriumbicarbonatproduktion. Diese Effekte auf die Magenschleimhaut sind innerhalb weniger Stunden nach Beendigung des Rauchens reversibel [18].

#### Magenkarzinome

**Figure 4:**
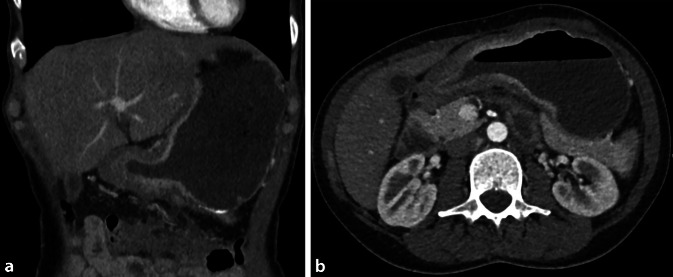

Das Magenkarzinom wird in bis zu 90 % aller Fälle durch eine Infektion mit *H. pylori* ausgelöst. Zusätzliches Rauchen steigert das Risiko deutlich [17]. In Abhängigkeit vom Alter erhöht sich das Risiko für die Entstehung von Magenkarzinomen bei Rauchern bis auf das 2,4Fache und bei Raucherinnen bis auf das 2,1Fache und steigt mit dem Alter zunehmend an (Abb. 4; [9]). Nach Beendigung des Rauchens zeigt sich eine signifikante Risikoreduktion abhängig von der Dauer der Tabakabstinenz [19].

### Pankreas

#### Akute und chronische Pankreatitis

**Figure 5:**
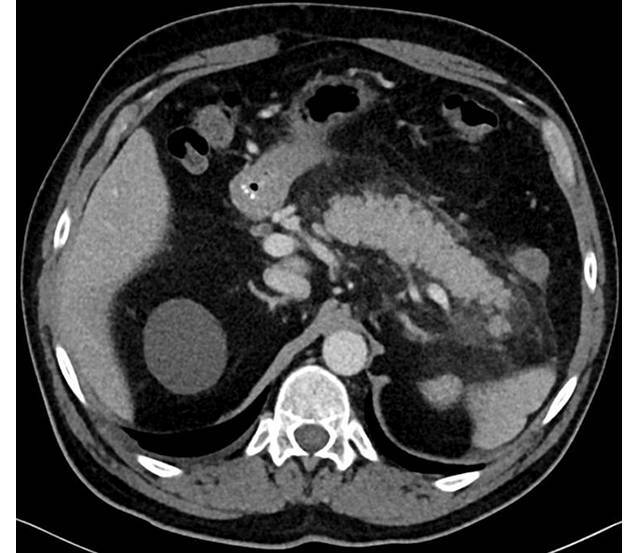

Unabhängig von Alkoholmissbrauch und Übergewicht besteht für aktive Raucher ein signifikant erhöhtes Risiko, eine akute oder chronische Pankreatitis zu entwickeln (Abb. 5). Es bleibt auch nach Raucherentwöhnung in geringerem Maß bestehen [20]. Das Risiko war in einer populationsbasierten dänischen Studie abhängig von den Pack Years, jedoch unabhängig von den beiden Hauptrisikofaktoren, dem Alkoholkonsum oder dem Gallensteinleiden [21]. Des Weiteren führt der Tabakkonsum zu einem rascheren Progress der Organveränderungen bei der alkoholischen chronischen Pankreatitis [22]. Die beiden Hauptmetaboliten des Tabakrauchs, Nikotin und NNK, führen zu funktionellen und histologischen Veränderungen des Pankreasparenchyms mit Auswirkungen auf die Azinuszellen, die duktale Sekretion sowie auf die Mikrogefäße [23].

#### Pankreaskarzinom

Bei der Entstehung des Pankreaskarzinoms spielen Lebensstilfaktoren eine wesentliche Rolle, wobei Tabakkonsum einen der relevantesten Risikofaktoren darstellt. Rauchende Personen weisen ein bis zu mehr als doppelt so hohes, mit zunehmendem Alter steigendes Risiko auf [9, 24]. Obwohl die genauen Mechanismen noch nicht bekannt sind, dürfte das Rauchen Auswirkungen auf Entzündung und Fibrose des Pankreasparenchyms haben. Diese sind wiederum die entscheidenden Charakteristika der chronischen Pankreatitis und somit bekannte Risikofaktoren für die Entwicklung eines Pankreaskarzinoms [23].

### Leber

#### Hepatozelluläres Karzinom

Das hepatozelluläre Karzinom (HCC) ist weltweit das fünfthäufigste Karzinom bei Männern und das siebthäufigste Karzinom bei Frauen – insgesamt ist es die dritthäufigste Ursache für krebsassoziierte Todesfälle. Neben bekannten Risikofaktoren, wie einer chronischen Hepatitis-B/C-Infektion, sind in westlichen Ländern vor allem Lebensstilfaktoren wie Alkoholabusus, Rauchen und Übergewicht relevant [25]. Altersabhängig wurde ein doppelt bis dreifach erhöhtes Risiko für die Entstehung von HCC bei Rauchern beschrieben [9]. Bei der Entstehung von Krebs hinterlassen die zahlreichen DNA-Mutationen typische Signaturen, also Narben im Genom. Wie auch in anderen Tumoren, wie z. B. der Lunge und dem Larynx, konnte beim HCC eine spezifische Tabaksignatur nachgewiesen werden, die direkt durch Tabakkarzinogene hervorgerufen wird [26].

#### Nichtalkoholische Fettlebererkrankung

Die nichtalkoholische Fettlebererkrankung („non-alcoholic fatty liver disease“, NAFLD) ist mittlerweile die am schnellsten zunehmende chronische Lebererkrankung mit einer weltweiten Prävalenz von 25–45 % [27]. Es gibt zunehmend Hinweise, dass Rauchen einen Einfluss auf die Entstehung einer NAFLD hat. So konnte in einer retrospektiven Analyse über einen Zeitraum von 10 Jahren gezeigt werden, dass Rauchen einen unabhängigen Risikofaktor für eine NAFLD darstellt. Des Weiteren gibt es einen signifikanten Zusammenhang zwischen Tabakrauchen und dem Schweregrad einer Leberfibrose [28]. Nikotin scheint hier einen additiven Effekt auf die durch eine hochkalorische, fettreiche Diät verursachte NAFLD mittels verschiedener Mechanismen zu haben. Es konnte gezeigt werden, dass Nikotin in Zusammenhang mit einer hochkalorischen, fettreichen Diät u. a. zu einem signifikant erhöhten oxidativen Stress, erhöhtem intrahepatischem Gehalt an Triglyceriden sowie zu einer gesteigerten Apoptose von Hepatozyten führt [28].

#### Primär sklerosierende Cholangitis

Das Risiko einer primär sklerosierenden Cholangitis (PSC) ist bei Rauchern und ehemaligen Rauchern signifikant niedriger als bei Nichtrauchern. Auch wenn nur Patienten mit PSC ohne entzündliche Darmerkrankung einbezogen werden, bleibt das Risiko im Vergleich zu Nichtrauchern deutlich niedriger. Ursächlich dürften die Auswirkungen von Nikotin auf die Immunabwehr mit einer reduzierten Wahrscheinlichkeit einer zellvermittelten Autoimmunität und somit einem geringeren Risiko einer PSC einhergehen [29].

## Darm

### Entzündliche Darmerkrankungen

#### Morbus Crohn

**Figure 6:**
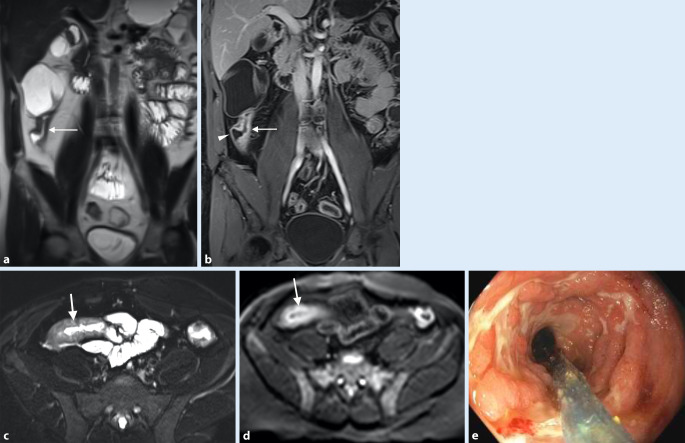

Von den bekannten modifizierbaren Lebensstilfaktoren ist Rauchen der wesentliche etablierte unabhängige Faktor für ein signifikant erhöhtes Risiko, einen M. Crohn zu entwickeln (5fach höher bei Frauen, 1,3fach höher bei Männern; Abb. 6). Es führt zu einem signifikant schlechteren Krankheitsverlauf mit einem erhöhten Risiko von Rezidiven sowie Hospitalisierungen. Des Weiteren benötigen die Patienten höhere Dosierungen immunsuppressiver Therapien und Biologika im Vergleich zu Nichtrauchern [30].

Risikofaktoren für ein postoperatives Rezidiv sind Alter, Lokalisation der Erkrankung und Rauchen, wobei Rauchen einen unabhängigen Risikofaktor mit einem bis zu zweifach erhöhten Risiko darstellt. Bei einem vollständigen postoperativen Rauchstopp konnten das Rezidivrisiko und die Notwendigkeit einer Reoperation signifikant reduziert werden [31]. Endoskopische Rezidive und die Entwicklung neuer Läsionen in vormals nicht betroffenen Darmsegmenten treten im ersten postoperativen Jahr bei 80 % der Patienten auf, wenn keine präventiven Maßnahmen, wie beispielsweise eine Raucherentwöhnung, getroffen werden [32].

Studien haben ergeben, dass bei Patienten mit M. Crohn eine mikrobielle Dysbiose des Darms mit einer Verringerung der mikrobiellen Vielfalt gefunden wird, welche die Integrität des Darmepithels beeinträchtigen und dadurch eine verstärkte Immunreaktion mit einer chronischen Entzündung auslösen kann. Eine gehäufte Koinzidenz von M. Crohn und chronisch-obstruktiver Lungenerkrankung (COPD) zeigt die Zusammenhänge zwischen Darm und Lunge und die gemeinsame Anfälligkeit für eine Schädigung des Mikrobioms durch Umweltreize wie Zigarettenrauch [4].

#### Colitis ulcerosa

Rauchen scheint einen protektiven Effekt auf die Entwicklung einer Colitis ulcerosa zu haben, mit einem bis zu 42 % niedrigeren Risiko im Vergleich zu Nichtrauchern [33]. Bei Patienten mit bereits diagnostizierter Colitis ulcerosa sind die Studienergebnisse hinsichtlich des Einflusses des Rauchens auf den Schweregrad und den Verlauf der Erkrankung divergent. In einer umfangreichen Kohortenstudie konnte zwischen Rauchern, ehemaligen Rauchern und Patienten, die nie geraucht hatten, kein signifikanter Unterschied bezüglich des Risikos von Krankheitsschüben nach Beendigung einer Kortisontherapie, der Häufigkeit einer notwendigen Kortisongabe, Hospitalisierung oder Kolektomien nachgewiesen werden. Es fanden sich auch keine Unterschiede zu Patienten, die innerhalb der ersten beiden Jahre nach Diagnosestellung aufhörten zu rauchen [34].

Die unterschiedlichen Auswirkungen des Rauchens auf Patienten mit M. Crohn und mit Colitis ulcerosa bleiben weiterhin ungeklärt, könnten aber auf die verschiedenen Folgen des Tabakrauchs auf den Dünn- und Dickdarm zurückzuführen sein [4].

#### Kolorektale Karzinome

**Figure 7:**
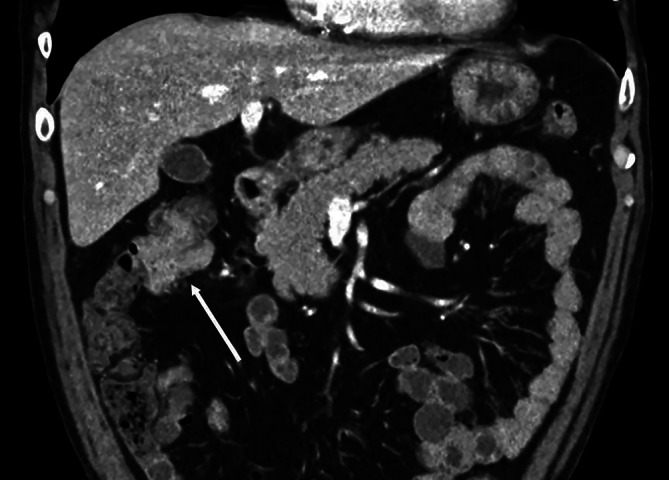

Raucher haben ein bis zu 2,4fach erhöhtes Risiko für die Entstehung von kolorektalen Karzinomen (Abb. 7), wobei das höchste Risiko in der Altersgruppe von 65–74 Jahren bei Männern und bei Frauen in der Altersgruppe von 55–74 Jahren besteht [9]. Dabei findet sich ein deutlicher Zusammenhang des Karzinomrisikos mit der Intensität und der Dauer des Rauchens. Die Beendigung des Rauchens senkt das Risiko für kolorektale Karzinome. Allerdings bleibt es wie auch bei anderen Karzinomen (z. B. Lunge und Ösophagus) weiterhin deutlich erhöht, selbst nach einem Rauchstopp vor 20 Jahren. Erst nach 25-jähriger Rauchabstinenz zeigt sich eine signifikante Reduktion des Dickdarmkrebsrisikos [35].

Kolorektale Adenome und serratierte Polypen sind benigne Vorstufen kolorektaler Karzinome. Raucher haben ein signifikant erhöhtes Risiko für die Entstehung kolorektaler Adenome mit einer klaren Dosis-Wirkungs-Beziehung und einem größeren Effekt bei Männern. Im Gegensatz zu Frauen erhöht sich das Risiko für die Adenomentstehung auch bei einer kürzeren Raucherhistorie (< 15 pack years). Ebenso verdoppelt sich das Risiko für das Auftreten von serratierten Polypen [36].

#### Zöliakie

Raucher weisen im Vergleich zu Menschen, die niemals geraucht haben, ein signifikant geringeres Risiko für die Entwicklung einer Zöliakie auf. Es besteht allerdings kein Unterschied zwischen ehemaligen Rauchern und Nie-Rauchern. Zugrundeliegende Ursachen für die protektive Wirkung des Zigarettenrauchs dürften immunmodulatorische Auswirkungen auf die zelluläre und humorale Immunfunktion und die reduzierte Durchlässigkeit des Darms sein [37].

#### Pouchitis

Es existieren unterschiedliche Studienergebnisse zu den Auswirkungen von Tabakrauchen auf das Risiko einer akuten oder chronischen Pouchitis nach ileoanaler Anastomose, meist bei Patienten mit Colitis ulcerosa. Eine Metaanalyse von Kani et al. hat ergeben, dass Rauchen weder einen schädigenden noch einen schützenden Effekt auf die Entwicklung einer Pouchitis hat [38].

## Diskussion

Tabakrauchen setzt Patienten einem signifikanten Risiko für verschiedene Erkrankungen des Gastrointestinaltrakts aus. Raucherassoziierte gastrointestinale Pathologien weisen allerdings in der radiologischen Bildgebung keine für Raucher spezifischen morphologischen Merkmale auf. Die Kenntnis einer Raucheranamnese sowie das Wissen um die Auswirkungen des Tabakrauchs auf gastrointestinale Erkrankungen können für eine optimale radiologische Diagnostik in dieser Patientengruppe hilfreich sein. Die Mechanismen, die zu einer Schädigung des Gastrointestinaltrakts führen, sind bisher unzureichend bekannt. Es bedarf weiterer Forschung, um die organspezifischen Auswirkungen des Tabakrauchens besser zu verstehen.

## Fazit für die Praxis

- Nikotin hat signifikante Auswirkungen auf den gesamten Gastrointestinaltrakt, die zu neoplastischen und entzündlichen Prozessen führen können.
- Tabakrauch verändert das intestinale Mikrobiom, das auch mit dem Respirationstrakt in Wechselwirkung stehen dürfte.
- Nikotinassoziierte gastrointestinale Erkrankungen weisen in der radiologischen Bildgebung keine für Raucher spezifischen morphologischen Merkmale auf.
- Die Kenntnis einer Raucheranamnese kann bei der diagnostischen Entscheidungsfindung hilfreich sein.
